# Astrocyte Activation in Locus Coeruleus Is Involved in Neuropathic Pain Exacerbation Mediated by Maternal Separation and Social Isolation Stress

**DOI:** 10.3389/fphar.2017.00401

**Published:** 2017-06-28

**Authors:** Kazuo Nakamoto, Fuka Aizawa, Megumi Kinoshita, Yutaka Koyama, Shogo Tokuyama

**Affiliations:** ^1^Department of Clinical Pharmacy, School of Pharmaceutical Sciences, Kobe Gakuin UniversityKobe, Japan; ^2^Laboratory of Pharmacology, Faculty of Pharmacy, Osaka Ohtani UniversityOsaka, Japan

**Keywords:** early life stress, astrocyte, glia, locus coeruleus, maternal separation and social isolation stress

## Abstract

Our previous studies demonstrated that emotional dysfunction associated with early life stress exacerbated nerve injury-induced mechanical allodynia. Sex differences were observed in several anxiety tests, but not in mechanical allodynia. To elucidate the mechanism underlying these findings, we have now investigated the involvement of astrocytes in emotional dysfunction and enhancement of nerve injury-induced mechanical allodynia in mice subjected to maternal separation combined with social isolation (MSSI) as an early life stress. We measured expression of glial fibrillary acidic protein (GFAP), an astrocyte maker, in each brain area by immunohistochemistry. GFAP expression in the locus coeruleus (LC) of female, but not of male mice, significantly increased after MSSI, corresponding to the behavioral changes at 7 and 12 weeks of age. Lipopolysaccharide (LPS)-treated astrocyte-derived supernatant was administered to local brain regions, including LC. Intra-LC injection of conditioned medium from cultured astrocytes treated with LPS increased GFAP expression, anxiety-like behavior and mechanical allodynia in both male and female mice. Furthermore, increases in anxiety-like behavior correlated with increased mechanical allodynia. These findings demonstrate that emotional dysfunction and enhanced nerve injury-induced mechanical allodynia after exposure to MSSI are mediated, at least in part, by astrocyte activation in the LC. Male but not female mice may show resistance to MSSI stress during growth.

## Introduction

Pain is a complex experience that involves purely sensory aspects, such as nociceptive stimuli, as well as emotional effects and feelings of unease (Bushnell et al., 2013). While pain serves as a warning against noxious stimuli (Woolf, 2010), chronic pain severely decreases quality of life. Also, emotional states heavily influence pain (Villemure and Bushnell, 2009). The severity of psychopathology in patients with major depressive disorder was positively associated with the prevalence of pain (Kishi et al., 2015). Thus, pain is modulated by both sensory and emotional factors. We previously demonstrated that emotional states altered mechanical allodynia and behavioral responses in mice (Nishinaka et al., 2015b). Another report showed that exposure to restraint stress before induction of neuropathic pain significantly increased mechanical allodynia (Norman et al., 2010). To obviate hyperalgesic effects associated directly with a physical stressor, we chose to use the maternal separation and social interaction (MSSI) stress model, a purely psychological form of stress. The MSSI model shows long-lasting behavioral and pathophysiological impairments (Niwa et al., 2011). We previously indicated that MSSI exacerbated mechanical allodynia. Interestingly, mice showed sex differences in several anxiety tests following MSSI, while pain was observed in both sexes after MSSI (Nishinaka et al., 2015b). These findings suggested that differences in sex may influence the relationship between pain control and emotional states.

Accumulating evidence indicates that astrocytes play a crucial role in the regulation of neural function in the central nervous system (CNS). Astrocytes are the most numerous type of glial cell. (Sofroniew and Vinters, 2010) and are important regulators of neural activity. For example, they contribute to neurotransmitter uptake and the CNS vascular response to neural activity (Sattler and Rothstein, 2006; Gordon et al., 2007). Moreover, astrocytes alter neural excitability via direct release of neuromodulator molecules (Halassa et al., 2009). In fact, astrocytes can release gliotransmitters (e.g., glutamate, GABA, D-serine, ATP) in response to neuronal activity including increase in the intracellular Ca^2+^ concentration (Araque et al., 2014; Gundersen et al., 2015). Exocytotic release of gliotransmitters or channel-mediated release via Ca^2+^-dependent Cl^-^ channel and volume-regulated anion channel (Kimelberg et al., 2006; Park et al., 2013) were induced by increase in the cytosolic Ca^2+^ concentration. Furthermore, gliotransmitters can also be released through Ca^2+^-independent opening of P2X purinergic receptors (Duan et al., 2003) or connexin/pannexin such as Cx43 hemichannels (Montero and Orellana, 2015). These factors can act on receptors on the presynaptic nerve terminal, or on post-synaptic dendrites when gliotransmitters are released from astrocytes. Astrocytes also contribute to the clinical and pathological mechanisms of disease processes. In a chronic pain study, reactive transformation of astrocytes following neuropathic pain contributed to the enhancement of neuropathic pain by releasing growth factors and inflammatory mediators (Zhang et al., 2013). Further, an astrocyte specific intermediate filament is up-regulated after nerve injury (Wei et al., 2008) and matrix metalloproteinase (MMP)-2-cleaved interleukin (IL)-1β induced astrocyte activation in the later phase of neuropathic pain (Kawasaki et al., 2008). More recently, glucocorticoid regulation of ATP release from spinal astrocytes underlies diurnal exacerbation of neuropathic mechanical allodynia (Koyanagi et al., 2016). Based on these reports, it is thought that astrocytes may play a role in modulation of pain.

On the other hand, astrocytes may influence neural networks that underlie specific behaviors, including aberrant behaviors associated with stress. For example, Cui et al. (2014), showed that glial dysfunction causes depressive-like behaviors and sleep disturbance. Chronic restraint stress causes dysfunction of astrocytes (Imbe et al., 2013). Zhang et al. (2015), showed that Chronic corticosterone exposure reduces hippocampal astrocyte structural plasticity in mice. A postmortem study reported that levels of glial fibrillary acidic protein (GFAP), a marker of reactive astrocytes, decreased in multiple brain regions in mood disorder patients (Miguel-Hidalgo et al., 2000; Bowley et al., 2002; Banasr and Duman, 2008; Chocyk et al., 2011; Gittins and Harrison, 2011). Although the mechanisms underlying emotional dysfunction mediated by astrocytes are unclear, these findings suggest that astrocytic abnormalities are associated with stress. In addition, astrocytes are regulated by sex hormones (Schwarz and Bilbo, 2012), indicating that there are sex differences in astrocytic function. We hypothesized that astrocytes are involved in sex-dependent emotional dysfunction induced by MSSI.

To examine our hypothesis, we investigated the influence of MSSI on GFAP expression levels and associated nociception and emotion in neuropathic pain states. Furthermore, to evaluate the involvement of local astrocytic activation, we developed a local activation model involving microinjection of supernatant from lipopolysaccharide (LPS)-treated astrocytes.

## Materials and Methods

### Animals

Pregnant ddY mice at gestational day 14 and timed-pregnant Wister rats (day 16 or 17) and were obtained from Japan SLC, Inc. (Hamamatsu, Japan). The pregnant mice and rat were housed individually under standard conditions (23–24°C, 12 h light/dark cycle with lights on from 8 a.m. to 8 p.m.) with food and water available ad libitum. The present study was conducted in accordance with the Guiding Principles for the Care and Use of Laboratory Animals adopted by the Japanese Pharmacological Society. The Ethical Committee for Animal Experimentation of Kobe Gakuin University approved all experiments (approval number A15-33; Kobe, Japan).

### Maternal Separation Combined with Social Isolation Stress Paradigm

Maternal separation combined with social isolation was performed as previously described (Nishinaka et al., 2015b). Pups both MSSI and control groups were housed with dams until postnatal day 14. On postnatal day 15, the pups in the MSSI group were placed in individual isolation cages (25 cm × 15 cm × 13 cm) for 6 h/day. The isolation cage was surrounded by the black paper to shut out visualization of other. After maternal separation for 7 days, pups were kept in isolation cages until 12 weeks of age. Dams and pups assigned to the control group were reared in standard conditions without maternal separation until weaning. After weaning, the control pups were separated by sex and housed 2–4 per cage.

### Elevated Plus-Maze Test

The elevated plus-maze (EPM) test was performed as previously described (Nishinaka et al., 2015b). Mice were placed on the EPM consisted of two open arms and two enclosed arms (both 25 cm length × 8 cm width) which elevated 50 cm above the floor. The illumination levels of the open and enclosed arms were similar (approximately 360 lx). The behavior was tracked for 5 min by using a web camera. The number of entries to the open arms was expressed as a percentage of enties to all arms. The time spent in the open arms was expressed as a percentage of times in the open arms. A decreased the number of entry and the time spent to the open arms indicaeted anixis-behavior (Nishinaka et al., 2015b).

### Partial Sciatic Nerve Ligation (PSL)

Mice were deeply anesthetized with sodium pentobarbital (65 mg/kg), and then surgery was performed as previously described at 9 weeks of age (Seltzer et al., 1990). In brief, the sciatic nerve of the right hind limb was exposed through a small incision. Half or two-thirds of the nerve thickness was tightly ligated with a silk suture. In sham-operated mice, the sciatic nerve was exposed without ligation.

### von Frey Test

Mechanical allodynia after nerve injury was measured using the von Frey test as previously described (Nishinaka et al., 2015b). Mice were placed on a 5 mm × 5 mm wire mesh grid floor for 2–3 h prior to testing. The middle of the plantar surface of each hind paw was probed with 0.4 g von Frey filaments (Neuroscience, Inc., Tokyo, Japan). The withdrawal response to probing of the hind paw was measured 10 times. The intertrial interval was >10 s. An increase in the number of withdrawals indicates the degree of pain associated with mechanical stimulation (Nishinaka et al., 2015b).

### Immunoflurorescence

Immunofluorescence staining was performed as previously described (Nakamoto et al., 2015) with some modifications. Mice were deeply anesthetized with diethyl ether and perfused transcardially with phosphate-buffered saline (PBS, pH 7.4) followed by 4% paraformaldehyde in 0.1 M PBS, pH 7.4. After perfusion, brain sections were incubated in 10% sucrose at 4°C for 3 h, and were kept 20% sucrose at 4°C overnight. Sections were cut at 20 μm on a cryostat (CM1850, Leica, Microsystems GmbH, Wetzlar, Germany). The brain sections were incubated with blocking buffer (3% BSA in PBST) for 1 h at room temperature, and then incubated overnight at 4°C with a mouse monoclonal anti-GFAP antibody (MAB3402, Merck Millipore KGaA, Darmstadt, Germany; 1:1000) and chicken polyclonal anti- tyrosine hydroxylase (TH) (Abcam, Tokyo, Japan; 1:200). The slices were incubated at room temperature for 2 h in secondary antibody (goat polyclonal anti-mouse IgG conjugated with AlexaFluor 488 or 594, goat polyclonal anti-chicken IgG conjugated with AlexaFluor 594; Life Technologies, Inc., Carlsbad, CA, United States; 1:200). The positive cells were detected with a confocal fluorescence microscope (FV1000, Olympus Corporation, Tokyo, Japan) or BZ-X710 microscope (Keyence, Itasca, IL, United States). Reactive astrocytes are characterized by cellular hypertrophy, hyperplasia, immunoreactivity of increased GFAP on tissue slides. The immunoreactivity of GFAP-positive astrocytic cells were quantified with the Image J cell counter analysis tool (ImageJ; NIH, Bethesda, MD, United States) in defined area of interest on the locus coeruleus (LC). The score was blinded to sampling times and animal treatments.

### Preparation of Primary Cultured Astrocytes from Rat Brain

Astrocytes were prepared from the cerebra of 1- to 2-day-old Wistar rats as described (Koyama et al., 2004). The isolated cells were seeded at 1 × 10^4^ cells/cm^2^ in 75 cm^2^ culture flasks and grown in minimal essential medium supplemented with 10% fetal bovine serum. To remove small process-bearing cells (mainly oligodendrocyte progenitors and microglia from the protoplasmic cell layer), the culture flasks were shaken at 250 rpm overnight 10–14 days after seeding. The monolayer cells were trypsinized and seeded on six-well culture plates or on 15 mm glass cover slips in 24-well culture plates. At this stage, approximately 95% of the cells showed immunoreactivity for GFAP.

### Lipopolysaccharide (LPS) Stimulation of Rat Astrocytes

Before treatment, astrocytes in six well-culture plates were incubated in serum-free medium for 48 h. LPS was minimally diluted using serum-free medium before treatment. After incubation in serum-free medium, the cells were treated with 1,000 ng/mL LPS for 24 h.

### Microinjection of Astrocyte Supernatant

Microinjection of astrocyte culture supernatant into the LC was performed as previously described (Nakamoto et al., 2015), with some modifications. Briefly, mice were anesthetized with pentobarbital (65 mg/kg) and immobilized on a stereotaxic frame (SR-5M; NARISHIGE, Co., Ltd, Tokyo, Japan). A microsyringe with a 30-gauge stainless steel needle was inserted unilaterally into the LC (5.4 mm posterior to bregma, 0.9 mm lateral from the midline, and 4.0 mm deep) and astrocyte supernatant (0.2 μL) injected incrementally over 1 min (**Figure 3A**). The injection site in LC was confirmed using 0.5% Trypan blue in saline.

### Statistical Analyses

All data are expressed as the mean ± the standard error of the mean (SEM). Significant differences were determined by a one-way analysis of variance (ANOVA) followed by Scheffe’s multiple comparison tests (for comparisons between more than three groups) or Student’s *t*-test (for comparisons between two groups). A *P*-value < 0.05 was considered significant.

## Results

### MSSI Stress Induced an Increase in GFAP Protein Expression in the LC Area of Female But Not Male Mice

**FIGURE 1:**
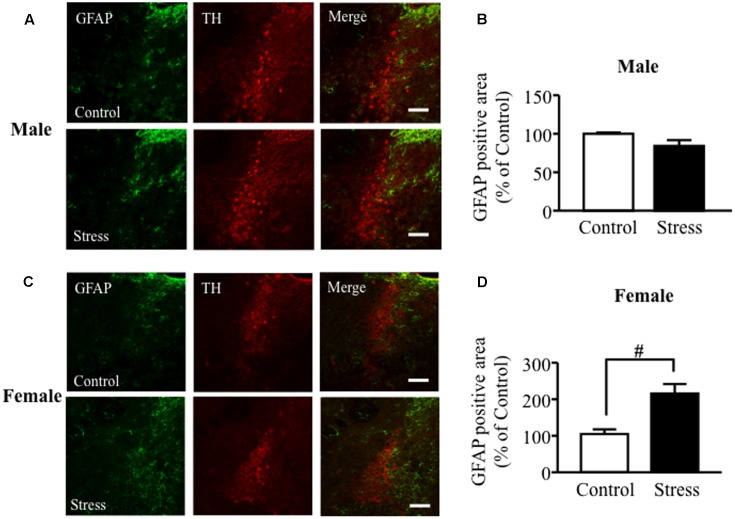
Maternal separation combined with social isolation (MSSI) stress induced an increase in GFAP protein expression in the LC area of female but not male mice. Representative images showing immunofluorescence labeling of GFAP (green) and TH (Red) in the LC of MSSI stressed mice at 7 weeks of age, before PSL. Percentage of the GFAP positive area over the total area including LC after MSSI in **(A,B)** male mice and **(C,D)** female mice. Panel shows representative images of the GFAP positive area. Results expressed as percent of Control (mean ± SEM) from *n* = 3 male mice and *n* = 3 female mice. ^#^*P* < 0.05, vs. Control, Student’s *t*-test. Scale bar = 100 mm. LC, locus coeruleus.

Immunoreactivity for GFAP was observed surrounding TH positive cells in the LC. In males, MSSI stress did not affect GFAP expression in the LC at 7 weeks of age, before PSL (**Figures 1A,B**). In contrast, MSSI stress induced a significant increase in GFAP expression in the LC of female mice, compared to control female mice, at 7 weeks of age (**Figures 1C,D**).

### The MSSI-Induced Increase in LC GFAP Expression Was Suppressed by PSL in Female Mice

**FIGURE 2:**
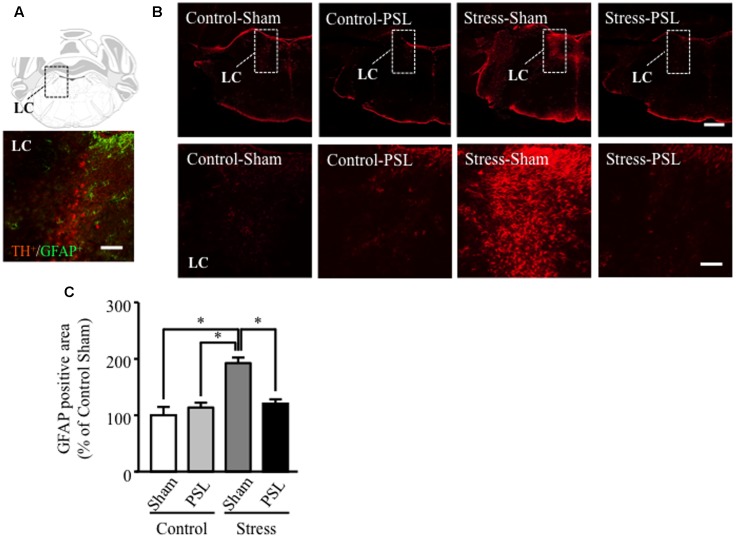
The MSSI-induced increase in LC GFAP expression was suppressed by PSL in female mice. Representative images showing immunofluorescence labeling of GFAP (green) and TH (Red) in the LC of mice **(A)**. Percentage of the GFAP positive area over the total area including LC of MSSI stressed mice at 12 weeks of age, after PSL after PSL **(B)**. Panel shows representative images of the GFAP positive area. Results expressed as percent of Control (mean ± SEM in female mice: *n* = 3) **(C)**. ^∗^*P* < 0.01 vs. Control-Sham, vs. Control-PSL, vs. Stress-PSL, Scheffe’s test. Scale bar = 100 mm. LC, locus coeruleus.

At 12 weeks of age, the increased GFAP protein expression was observed in brain regions including the LC of MSSI-stressed female mice, compared to control female mice. This MSSI-induced increase in LC GFAP expression was suppressed by PSL (**Figures 2A,B**). In other region of the brain, GFAP protein expression did not change in male and female mice (Supplementary Figure 1).

### LPS-Treated Astrocyte-Conditioned Medium Microinjection into the LC Induced Increased GFAP Expression in Male and Female Mice

**FIGURE 3:**
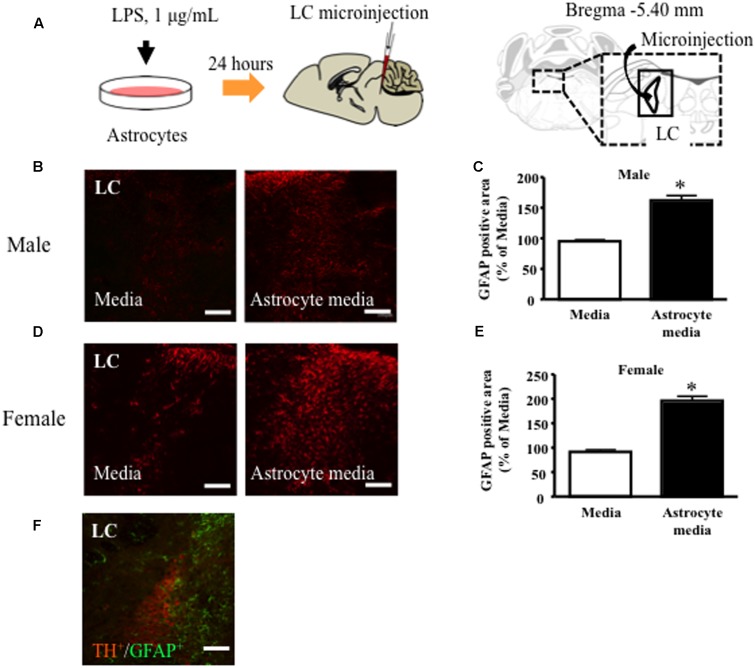
Lipopolysaccharide (LPS)-treated astrocyte-conditioned medium microinjection into the LC increased GFAP expression in male and female mice. **(A)** Schematic diagram of the experimental protocol for microinjection into the LC. LC, locus coeruleus. Representative images showing immunofluorescence labeling of GFAP (red) in the LC of mice. **(A)** Percentage of the GFAP positive area over the total area including LC after microinjection in male **(B,C)** and female **(D,E)** mice. GFAP (Red) ^∗^*P* < 0.05, vs. Media, Student’s *t*-test. Representative images showing immunofluorescence labeling of GFAP (green) and TH (Red) in the LC of mice **(F)**. Representative images of astrocytes in LC after microinjection. GFAP (Green), TH (Red). Scale bar = 100 mm.

In male and female mice, microinjection of LPS-treated astrocyte-derived supernatant into the brainstem area, including the LC, significantly increased local GFAP expression compared to non-treated control medium (**Figures 3B–E**). GFAP positive cells were observed surrounding TH positive cells in the LC (**Figure 3F**).

### LPS-Treated Astrocyte-Conditioned Medium Microinjection into the LC Induced Anxiety-Like Behavior and PSL-Induced Mechanical Allodynia in Male Mice

**FIGURE 4:**
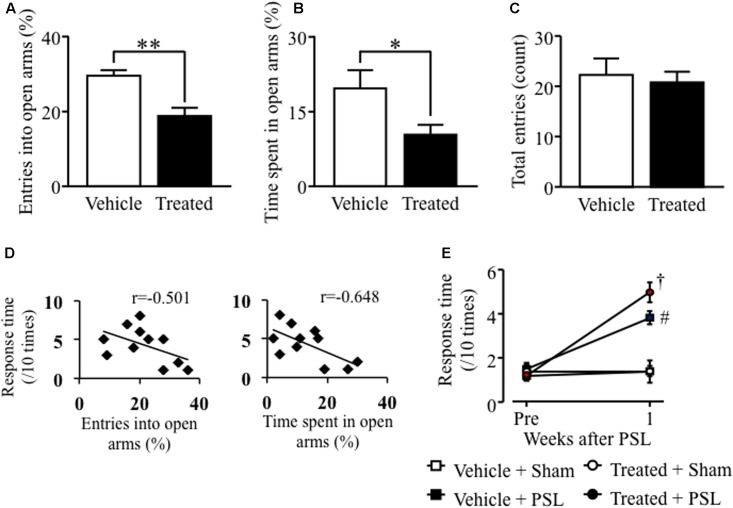
Lipopolysaccharide-treated astrocyte-conditioned medium microinjection into the LC induced anxiety-like behavior and PSL-induced mechanical allodynia in male mice. **(A)** The number of entries into open arms (%). **(B)** Time spent in the open arms (%). **(C)** The total number of entries into the four arms. Vehicle group: *n* = 8, Treated group: *n* = 9. **(D)** Correlation between the entries into open arms (%) or the time spent in open arms (%) and mechanical stimuli after PSL in vehicle- or LPS-treated male mice. **(E)** 0.4 g filament. Vehicle-Sham group: *n* = 5, Vehicle-PSL group: *n* = 6, Treated-Sham group: *n* = 5, Treated-PSL group: *n* = 11. Data expressed as the mean ± SEM. ^∗^*P* < 0.05, ^∗∗^*P* < 0.01 vs. Vehicle, Student’s *t*-test. ^#^*P* < 0.05 vs. Vehicle + Sham, ^†^*P* < 0.05 vs. Treated + Sham, Scheffe’s test.

In male mice, microinjection of LPS-treated astrocyte-derived supernatant into the brainstem area, including the LC, significantly decreased the number of open arm crossings and time spent in the open arms compared to vehicle-treated male mice (**Figures 4A,B**). In contrast, there was no difference in the number of center zone crossings between vehicle- and LPS-treated groups (**Figure 4C**). The entries into open arms (%) or the time spent in open arms (%) correlated negatively with the response time to mechanical stimuli after PSL in vehicle- or LPS-treated male mice (**Figure 4D**). At 1 week after PSL, the vehicle- and LPS-treated astrocyte-derived supernatant treated mice showed an increased number of responses to mechanical stimuli compared to vehicle- and LPS-treated injected sham injured male mice. Mice injected with LPS-treated astrocyte-derived supernatant showed a tendency toward an increase in the number of responses to mechanical stimuli compared to vehicle-treated injected PSL male mice. However, the number of responses against mechanical stimuli did not change between the vehicle treated group and the LPS-treated astrocyte-derived supernatant group prior to PSL (**Figure 4E**).

### LPS-Treated Astrocyte-Derived Supernatant Microinjection into the LC Caused Anxiety-Like Behavior and PSL-Induced Mechanical Allodynia in Female Mice

**FIGURE 5:**
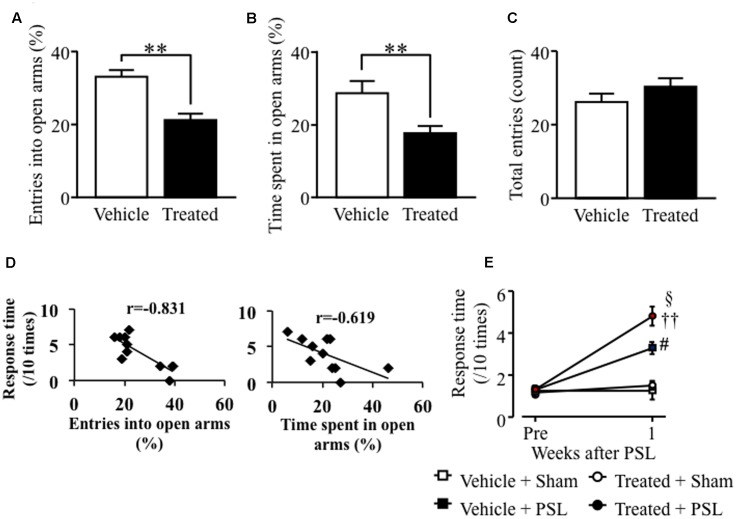
Lipopolysaccharide-treated astrocyte-derived supernatant microinjection into the LC caused anxiety-like behavior and PSL-induced mechanical allodynia in female mice. **(A)** The number of entries into open arms (%). **(B)** Time spent in the open arms (%). **(C)** The total number of entries into the four arms. Vehicle group: *n* = 8, Treated group: *n* = 11. **(D)** Correlation between the entries into open arms (%) or the time spent in open arms (%) and mechanical stimuli after PSL in vehicle- or LPS-treated female mice. **(E)** 0.4 g filament. Vehicle-Sham group: *n* = 4, Vehicle-PSL group: *n* = 7, Treated-Sham group: *n* = 6, Treated-PSL group: *n* = 10. Data expressed as mean ± SEM. ^∗∗^*P* < 0.01 vs. Vehicle, Student’s *t*-test. ^#^*P* < 0.05 vs. Vehicle + Sham, ^††^*P* < 0.01 vs. Treated + Sham, ^x^*P* < 0.05 vs. Treated + PSL, Scheffe’s test.

Similar results were obtained in female mice. Female mice microinjected with LPS-treated astrocyte-derived supernatant showed a decreased number of open arm crossings and time spent in the open arms compared to vehicle-treated female mice (**Figures 5A,B**). The number of crossings in the center zone was comparable to that in vehicle-treated female mice (**Figure 5C**). The entries into open arms (%) or the time spent in open arms (%) correlated negatively with the response time to mechanical stimuli after PSL in vehicle- or LPS-treated female mice (**Figure 5D**). At 1 week after PSL, vehicle- and LPS-treated astrocyte-derived supernatant female mice showed an increased number of responses to mechanical stimuli compared to vehicle- and LPS-treated supernatant- injected female mice. Furthermore, the number of responses to mechanical stimuli showed a significant increase in LPS-treated PSL female mice compared to vehicle-treated PSL female mice. In contrast, the number of responses to mechanical stimuli before PSL was similar in both vehicle- and LPS-treated supernatant-injected female mice (**Figure 5E**).

## Discussion

Both the LC in the pons and the rostral ventromedial medulla (RVM) contribute to the regulation of pain and emotion (Aston-Jones et al., 1999; Yoshimura and Furue, 2006). Noradrenergic and serotonergic neurons are localized to the LC and RVM, respectively, and project to multiple higher brain areas as well as to the spinal cord. The descending pathways regulate nociceptive signaling in the spinal cord. It has been reported that chronic stress activates or suppress neuronal activity in the LC, and neuropathic pain suppress neuronal activity in the LC, which is associated with the development of anxiety-like behaviors in mice (Borges et al., 2015). Selective depletion of noradrenaline in the LC causes anxiety- and depression-like behaviors (Itoi et al., 2011). Physiological changes in glial cells of the medulla oblongata are associated with disease states and altered neuronal activity (Alvarez-Maubecin et al., 2000; O’Donnell et al., 2012). For example, downregulation of astrocytic gene expression was observed in the postmortem LC of major depressive disorder patients (Chandley et al., 2013). Chronic restraint stress decreased the expression of GFAP and S100β calcium-binding protein in the cytoplasm of astrocytes (Gerlai et al., 1995) in the RVM (Imbe et al., 2013), while inflammation-induced reactive astrocytes enhanced the activity of the serotonergic descending pain facilitation system, which increased nociceptive signaling in the spinal cord via the RVM (Cunha and Dias, 2009; Roberts et al., 2009). In this study, we examined the influence of MSSI on astrocytes in the LC to determine whether astrocytes were involved in the exacerbation of neuropathic pain by emotional dysfunction. We found that MSSI significantly increased GFAP expression in the LC of female but not male mice. Furthermore, we found that GFAP expression in the medulla oblongata of MSSI stressed female mice shows tendency of increase at 12 weeks age, but not male mice (Supplementary Figures 1, 2). It is well-known that up-regulation of GFAP levels is associated with functional changes in astrocytes that in turn affect neuronal function, due to changes in astrocyte-neuron signaling (Gómez-Galán et al., 2013). Therefore, these findings suggest that MSSI-induced dysfunctional astrocytes in the LC are involved in the development of abnormal emotional states and alterations of the pain modulatory system.

Our previous results demonstrated that MSSI increased anxiety-like behaviors in female but not male mice (Nishinaka et al., 2015b) and MSSI did not influence GFAP expression in the LC of male mice. However, Niwa et al. (2011) previously found that MSSI increased EPM anxiety-like behavior in both male and female C57BL/6J mice. In mice, anxiety and depression-like behaviors differ according to strain or sex (Millstein and Holmes, 2007; McDermott et al., 2015). Generally, females have increased stress sensitivity, likely due to differences in sex hormones, although the detailed mechanisms are not clear (Maeng and Milad, 2015). As show in **Figures 1**, **2**, at 7 or 12 weeks age, the GFAP expression in the LC of MSSI stressed mice with or without PSL treated was different between male and female. These results indicate that MSSI-mediated emotional dysfunction and neuropathic pain exacerbation might be more sensitively induced or worked astrocyte activation in female stressed mice, but not male stressed mice. Therefore, our findings suggest that sex differences in LC astrocytic activation induced by MSSI are responsible, at least in part, for the sex differences in anxiety-like behavior and are mediated through the damaging effects of reactive astrocytes on female LC neurons.

Furthermore, to investigate the effect of acute and region-selective activation of LC astrocytes on anxiety-like or nociceptive behaviors, we microinjected the supernatant of LPS-treated astrocytes into the LC region. We found that LC astrocytes up-regulated GFAP expression and showed morphological changes, indicating that astrocytes were activated by the supernatant injection. LPS binds to toll like receptor (TLR) 4, which is expressed in astrocytes. It is thought that these signaling-induced inflammatory responses, which are mediated by the NF-κB pathway, may result in activation of astrocytes (Gorina et al., 2011; Li et al., 2016). Activated astrocytes show up-regulated GFAP protein levels and morphological changes, such as cellular hypertrophy and outgrowth of astrocytic foot processes (von Boyen et al., 2004; Pekny and Pekna, 2014). In this study, we used the LPS-treated astrocytes from rat cell culture. As shown in previous reports, co-culture systems have been used which combined rat astrocytes with mouse neurons. And also, it is reported that rat astrocytes provided optimum conditions for synaptic functioning of mouse neurons (Goudriaan et al., 2014). Further, it is reported that there was no cross-reactivity toward mouse-derived embryonic stem cells injected into rat retina (Gregory-Evans et al., 2009). In this study, microinjection of supernatant from vehicle-treated astrocytes was not affected GFAP expression in the LC area. Based on these reports, we believe that cross-reactivity which may be induced by the microinjection of supernatant from rat astrocytes into mice may have a negligible effect on GFAP protein expression.

The important point is that LC administration of supernatant derived from LPS-treated astrocytes increased anxiety-like behavior and GFAP expression in both male and female mice. These results indicate that activation of astrocytes in the LC might result in emotional dysfunction and exacerbated mechanical allodynia. However, the GFAP expression in the LC of MSSI mice with PSL was different between males and females. We previously showed that MSSI sex-dependently induced emotional dysfunction after nerve injury, which was associated with sex differences in the stress-induced regulation of BDNF expression (Nishinaka et al., 2015a). It is thought that neuronal function in the brain of male mice is protected from early life stresses, such as MSSI, by altered expression of several stress-responsive factors, including BDNF upregulation, such that MSSI male mice might acquire resistance to stress. Our findings suggest that activation of astrocytes in the LC, caused by MSSI, might be involved in the induction of emotional dysfunction.

We also found that LC astrocytic dysfunction caused by MSSI contributes to the exacerbation of mechanical allodynia after PSL. Despite downregulation of MSSI-induced LC GFAP overexpression by PSL, MSSI enhanced nerve injury-induced mechanical allodynia. Many studies on the relationship between pain and astrocytic function have demonstrated that inflammation or nerve injury activates astrocytes in the spinal cord and supraspinal area, and that these activated cells contribute to the development and maintenance of persistent pain (Ji et al., 2013). Previous studies have also shown that anti-inflammatory drugs, such as steroids, suppress activated astrocytes (Kuypers et al., 2013; Evans et al., 2014). The MSSI-induced increase in GFAP expression was reversed to control levels by PSL, which would appear to be an improvement in the MSSI effect. From our results, it is unclear whether PSL-induced downregulation of LC GFAP expression in MSSI-stressed mice is associated with the exacerbation of nerve injury-induced mechanical allodynia. We suggest that the MSSI-induced increase in LC GFAP expression in the absence of nerve injury may induce a reduction in mechanical threshold in the presence of nerve injury. The activated astrocytes produce various neurotrophic factors, cytokines, chemokines, and free radicals, with both neuroprotective and neurotoxic effects. The balance between these factors is important for preservation of neuronal function and may act to trigger a compensatory condition against MSSI. Nerve injury may shift this balance and thereby enhance mechanical allodynia.

Furthermore, as shown in **Figures 4**, **5**, we found that activation of astrocytes which induced by microinjection of astrocyte conditioned medium with LPS caused emotional dysfunction and exacerbated mechanical allodynia. On the other hand, there is no increased GFAP expression following MSSI in male mice although MSSI-mediated neuropathic pain exacerbation observed in male mice (Nishinaka et al., 2015a). This discrepancy between male and female indicates that other factors which inhibit astrocyte activation may be produced in MSSI stressed male mice, but not female stressed mice. Further studies will be needed to clarify sex difference between astrocyte activation and pain.

## Conclusion

We found that MSSI sex-dependently activated astrocytes in the LC, as indicated by increased GFAP expression, with increases in anxiety-like behavior. These MSSI-induced activated astrocytes in the LC may contribute to the exacerbation of neuropathic pain. Furthermore, male mice, but not female mice, might acquire resistance to MSSI-induced stress during growth. We have thus identified one possible and unexpected mechanism linking emotional state with changes in the pain control system, i.e., astroglial activation in the LC.

## Author Contributions

Study conception and design: KN and ST; acquisition of data: KN, MK, YK; analysis and interpretation: KN, MK and ST; drafting of the manuscript: KN and ST; critical revision of the manuscript for important intellectual content; ST, statistical analysis; KN, MK; obtained funding; KN, ST, administrative, technical, or material support; KN, MK, YK, study supervision; KN, ST. All authors read and approved the final manuscript.

## Conflict of Interest Statement

The authors declare that the research was conducted in the absence of any commercial or financial relationships that could be construed as a potential conflict of interest.

## References

[B1] Alvarez-Maubecin V. Garcia-Hernandez F. Williams J. T. Van Bockstaele E. J. (2000). Functional coupling between neurons and glia. *J. Neurosci.* 20 4091–4098. 10818144 10.1523/JNEUROSCI.20-11-04091.2000 PMC6772654

[B2] Araque A. Carmignoto G. Haydon P. G. Oliet S. H. Robitaille R. Volterra A. (2014). Gliotransmitters travel in time and space. *Neuron* 81 728–739. 10.1016/j.neuron.2014.02.007 24559669 PMC4107238

[B3] Aston-Jones G. Rajkowski J. Cohen J. (1999). Role of locus coeruleus in attention and behavioral flexibility. *Biol. Psychiatry* 46 1309–1320. 10.1016/S0006-3223(99)00140-7 10560036

[B4] Banasr M. Duman R. S. (2008). Glial loss in the prefrontal cortex is sufficient to induce depressive-like behaviors. *Biol. Psychiatry* 64 863–870. 10.1016/j.biopsych.2008.06.008 18639237 PMC2709733

[B5] Borges G. Berrocoso E. Mico J. A. Neto F. (2015). ERK1/2: Function, signaling and implication in pain and pain-related anxio-depressive disorders. *Prog. Neuropsychopharmacol. Biol. Psychiatry* 60 77–92. 10.1016/j.pnpbp.2015.02.010 25708652

[B6] Bowley M. P. Drevets W. C. Ongür D. Price J. L. (2002). Low glial numbers in the amygdala in major depressive disorder. *Biol. Psychiatry* 52 404–412. 10.1016/S0006-3223(02)01404-X 12242056

[B7] Bushnell M. C. Ceko M. Low L. A. (2013). Cognitive and emotional control of pain and its disruption in chronic pain. *Nat. Rev. Neurosci.* 14 502–511. 10.1038/nrn3516 23719569 PMC4465351

[B8] Chandley M. J. Szebeni K. Szebeni A. Crawford J. Stockmeier C. A. Turecki G. (2013). Gene expression deficits in pontine locus coeruleus astrocytes in men with major depressive disorder. *J. Psychiatry Neurosci.* 38 276–284. 10.1503/jpn.120110 23415275 PMC3692725

[B9] Chocyk A. Dudys D. Przyborowska A. Majcher I. Maćkowiak M. Wędzony K. (2011). Maternal separation affects the number, proliferation and apoptosis of glia cells in the substantia nigra and ventral tegmental area of juvenile rats. *Neuroscience* 173 1–18. 10.1016/j.neuroscience.2010.11.037 21108994

[B10] Cui W. Mizukami H. Yanagisawa M. Aida T. Nomura M. Isomura Y. (2014). Glial dysfunction in the mouse habenula causes depressive-like behaviors and sleep disturbance. *J. Neurosci.* 34 16273–16285. 10.1523/JNEUROSCI.1465-14.2014 25471567 PMC6608483

[B11] Cunha T. M. Dias Q. M. (2009). Glial modulation of pain: a step beyond. *J. Neurosci.* 29 3340–3342. 10.1523/JNEUROSCI.5938-08.2009 19295140 PMC6665261

[B12] Duan S. Anderson C. M. Keung E. C. Chen Y. Chen Y. Swanson R. A. (2003). P2X7 receptor-mediated release of excitatory amino acids from astrocytes. *J. Neurosci.* 23 1320–1328. 12598620 10.1523/JNEUROSCI.23-04-01320.2003 PMC6742264

[B13] Evans M. C. Gaillard P. J. de Boer M. Appeldoorn C. Dorland R. Sibson N. R. (2014). CNS-targeted glucocorticoid reduces pathology in mouse model of amyotrophic lateral sclerosis. *Acta Neuropathol. Commun.* 2:66 10.1186/2051-5960-2-66 PMC4229735 24923195

[B14] Gerlai R. Wojtowicz J. M. Marks A. Roder J. (1995). Overexpression of a calcium-binding protein, S100 beta, in astrocytes alters synaptic plasticity and impairs spatial learning in transgenic mice. *Learn. Mem.* 2 26–39. 10.1101/lm.2.1.26 10467564

[B15] Gittins R. A. Harrison P. J. (2011). A morphometric study of glia and neurons in the anterior cingulate cortex in mood disorder. *J. Affect. Disord.* 133 328–332. 10.1016/j.jad.2011.03.042 21497910

[B16] Gómez-Galán M. De Bundel D. Van Eeckhaut A. Smolders I. Lindskog M. (2013). Dysfunctional astrocytic regulation of glutamate transmission in a rat model of depression. *Mol. Psychiatry* 18 582–594. 10.1038/mp.2012.10 22371047

[B17] Gordon G. R. J. Mulligan S. J. MacVicar B. A. (2007). Astrocyte control of the cerebrovasculature. *Glia* 55 1214–1221. 10.1002/glia.20543 17659528

[B18] Gorina R. Font-Nieves M. Márquez-Kisinousky L. Santalucia T. Planas A. M. (2011). Astrocyte TLR4 activation induces a proinflammatory environment through the interplay between MyD88-dependent NFκB signaling, MAPK, and Jak1/Stat1 pathways. *Glia* 59 242–255. 10.1002/glia.21094 21125645

[B19] Goudriaan A. Camargo N. Carney K. E. Oliet S. H. Smit A. B. Verheijen M. H. (2014). Novel cell separation method for molecular analysis of neuron-astrocyte co-cultures. *Front. Cell Neurosci.* 8:12 10.3389/fncel.2014.00012 PMC3906515 24523672

[B20] Gregory-Evans K. Chang F. Hodges M. D. Gregory-Evans C. Y. (2009). Ex vivo gene therapy using intravitreal injection of GDNF-secreting mouse embryonic stem cells in a rat model of retinal degeneration. *Mol. Vis.* 15 962–973. 19461934 PMC2684563

[B21] Gundersen V. Storm-Mathisen J. Bergersen L. H. (2015). Neuroglial transmission. *Physiol. Rev.* 95 695–726. 10.1152/physrev.00024.2014 26084688

[B22] Halassa M. M. Fellin T. Haydon P. G. (2009). Tripartite synapses: roles for astrocytic purines in the control of synaptic physiology and behavior. *Neuropharmacology* 57 343–346. 10.1016/j.neuropharm.2009.06.031 19577581 PMC3190118

[B23] Imbe H. Kimura A. Donishi T. Kaneoke Y. (2013). Effects of restraint stress on glial activity in the rostral ventromedial medulla. *Neuroscience* 241 10–21. 10.1016/j.neuroscience.2013.03.008 23518226

[B24] Itoi K. Sugimoto N. Suzuki S. Sawada K. Das G. Uchida K. (2011). Targeting of locus ceruleus noradrenergic neurons expressing human interleukin-2 receptor α-subunit in transgenic mice by a recombinant immunotoxin anti-Tac(Fv)-PE38: a study for exploring noradrenergic influence upon anxiety-like and depression-like beha. *J. Neurosci.* 31 6132–6139. 10.1523/JNEUROSCI.5188-10.2011 21508238 PMC6632972

[B25] Ji R. R. Berta T. Nedergaard M. (2013). Glia and pain: is chronic pain a gliopathy? *Pain* 154(Suppl.), S10–S28. 10.1016/j.pain.2013.06.022 23792284 PMC3858488

[B26] Kawasaki Y. Xu Z. Z. Wang X. Park J. Y. Zhuang Z. Y. Tan P. H. (2008). Distinct roles of matrix metalloproteases in the early- and late-phase development of neuropathic pain. *Nat. Med.* 14 331–336. 10.1038/nm1723 18264108 PMC2279180

[B27] Kimelberg H. K. MacVicar B. A. Sontheimer H. (2006). Anion channels in astrocytes: biophysics, pharmacology, and function. *Glia* 54 747–757. 10.1002/glia.20423 17006903 PMC2556042

[B28] Kishi T. Matsuda Y. Mukai T. Matsunaga S. Yasue I. Fujita K. (2015). A cross-sectional survey to investigate the prevalence of pain in Japanese patients with major depressive disorder and schizophrenia. *Compr. Psychiatry* 59 91–97. 10.1016/j.comppsych.2015.02.004 25724075

[B29] Koyama Y. Yoshioka Y. Shinde M. Matsuda T. Baba A. (2004). Focal adhesion kinase mediates endothelin-induced cyclin D3 expression in rat cultured astrocytes. *J. Neurochem.* 90 904–912. 10.1111/j.1471-4159.2004.02546.x 15287896

[B30] Koyanagi S. Kusunose N. Taniguchi M. Akamine T. Kanado Y. Ozono Y. (2016). Glucocorticoid regulation of ATP release from spinal astrocytes underlies diurnal exacerbation of neuropathic mechanical allodynia. *Nat. Commun.* 7:13102 10.1038/ncomms13102 PMC5067584 27739425

[B31] Kuypers E. Jellema R. K. Ophelders D. R. M. G. Dudink J. Nikiforou M. Wolfs T. G. A. M. (2013). Effects of intra-amniotic lipopolysaccharide and maternal betamethasone on brain inflammation in fetal sheep. *PLoS ONE* 8:e81644 10.1371/journal.pone.0081644 PMC3866104 24358119

[B32] Li N. Zhang X. Dong H. Zhang S. Sun J. Qian Y. (2016). Lithium ameliorates LPS-induced astrocytes activation partly via inhibition of toll-like receptor 4 expression. *Cell Physiol. Biochem.* 38 714–725. 10.1159/000443028 26870942

[B33] Maeng L. Y. Milad M. R. (2015). Sex differences in anxiety disorders: interactions between fear, stress, and gonadal hormones. *Horm. Behav.* 76 106–117. 10.1016/j.yhbeh.2015.04.002 25888456 PMC4823998

[B34] McDermott C. M. Liu D. Ade C. Schrader L. A. (2015). Estradiol replacement enhances fear memory formation, impairs extinction and reduces COMT expression levels in the hippocampus of ovariectomized female mice. *Neurobiol. Learn. Mem.* 118 167–177. 10.1016/j.nlm.2014.12.009 25555360

[B35] Miguel-Hidalgo J. J. Baucom C. Dilley G. Overholser J. C. Meltzer H. Y. Stockmeier C. A. (2000). Glial fibrillary acidic protein immunoreactivity in the prefrontal cortex distinguishes younger from older adults in major depressive disorder. *Biol. Psychiatry* 48 861–873. 10.1016/S0006-3223(00)00999-9 11063981

[B36] Millstein R. A. Holmes A. (2007). Effects of repeated maternal separation on anxiety- and depression-related phenotypes in different mouse strains. *Neurosci. Biobehav. Rev.* 31 3–17. 10.1016/j.neubiorev.2006.05.003 16950513

[B37] Montero T. D. Orellana J. A. (2015). Hemichannels: new pathways for gliotransmitter release. *Neuroscience* 286 45–59. 10.1016/j.neuroscience.2014.11.048 25475761

[B38] Nakamoto K. Nishinaka T. Sato N. Aizawa F. Yamashita T. Mankura M. (2015). The activation of supraspinal GPR40/FFA1 receptor signalling regulates the descending pain control system. *Br. J. Pharmacol.* 172 1250–1262. 10.1111/bph.13003 25362997 PMC4337699

[B39] Nishinaka T. Kinoshita M. Nakamoto K. Tokuyama S. (2015a). Sex differences in depression-like behavior after nerve injury are associated with differential changes in brain-derived neurotrophic factor levels in mice subjected to early life stress. *Neurosci. Lett.* 592 32–36. 10.1016/j.neulet.2015.02.053 25725169

[B40] Nishinaka T. Nakamoto K. Tokuyama S. (2015b). Enhancement of nerve-injury-induced thermal and mechanical hypersensitivity in adult male and female mice following early life stress. *Life Sci.* 121 28–34. 10.1016/j.lfs.2014.11.012 25476827

[B41] Niwa M. Matsumoto Y. Mouri A. Ozaki N. Nabeshima T. (2011). Vulnerability in early life to changes in the rearing environment plays a crucial role in the aetiopathology of psychiatric disorders. *Int. J. Neuropsychopharmacol.* 14 459–477. 10.1017/S1461145710001239 20950517

[B42] Norman G. J. Karelina K. Zhang N. Walton J. C. Morris J. S. Devries A. C. (2010). Stress and IL-1beta contribute to the development of depressive-like behavior following peripheral nerve injury. *Mol. Psychiatry* 15 404–414. 10.1038/mp.2009.91 19773812 PMC5214062

[B43] O’Donnell J. Zeppenfeld D. McConnell E. Pena S. Nedergaard M. (2012). Norepinephrine: a neuromodulator that boosts the function of multiple cell types to optimize CNS performance. *Neurochem. Res.* 37 2496–2512. 10.1007/s11064-012-0818-x 22717696 PMC3548657

[B44] Park H. Han K. S. Oh S. J. Jo S. Woo J. Yoon B. E. (2013). High glutamate permeability and distal localization of Best1 channel in CA1 hippocampal astrocyte. *Mol. Brain* 6:54 10.1186/1756-6606-6-54 PMC4029177 24321245

[B45] Pekny M. Pekna M. (2014). Astrocyte reactivity and reactive astrogliosis: costs and benefits. *Physiol. Rev.* 94 1077–1098. 10.1152/physrev.00041.2013 25287860

[B46] Roberts J. Ossipov M. H. Porreca F. (2009). Glial activation in the rostroventromedial medulla promotes descending facilitation to mediate inflammatory hypersensitivity. *Eur. J. Neurosci.* 30 229–241. 10.1111/j.1460-9568.2009.06813.x 19614984 PMC5693227

[B47] Sattler R. Rothstein J. D. (2006). Regulation and dysregulation of glutamate transporters. *Handb. Exp. Pharmacol.* 175 277–303. 10.1007/3-540-29784-7_14 16722241

[B48] Schwarz J. M. Bilbo S. D. (2012). Sex, glia, and development: interactions in health and disease. *Horm. Behav.* 62 243–253. 10.1016/j.yhbeh.2012.02.018 22387107 PMC3374064

[B49] Seltzer Z. Dubner R. Shir Y. (1990). A novel behavioral model of neuropathic pain disorders produced in rats by partial sciatic nerve injury. *Pain* 43 205–218. 10.1016/0304-3959(90)91074-S 1982347

[B50] Sofroniew M. V. Vinters H. V. (2010). Astrocytes: biology and pathology. *Acta Neuropathol.* 119 7–35. 10.1007/s00401-009-0619-8 20012068 PMC2799634

[B51] Villemure C. Bushnell M. C. (2009). Mood influences supraspinal pain processing separately from attention. *J. Neurosci.* 29 705–715. 10.1523/JNEUROSCI.3822-08.2009 19158297 PMC2768393

[B52] von Boyen G. B. Steinkamp M. Reinshagen M. Schäfer K. H. Adler G. Kirsch J. (2004). Proinflammatory cytokines increase glial fibrillary acidic protein expression in enteric glia. *Gut* 53 222–228. 10.1136/gut.2003.012625 14724154 PMC1774931

[B53] Wei F. Guo W. Zou S. Ren K. Dubner R. (2008). Supraspinal glial-neuronal interactions contribute to descending pain facilitation. *J. Neurosci.* 28 10482–10495. 10.1523/JNEUROSCI.3593-08.2008 18923025 PMC2660868

[B54] Woolf C. J. (2010). What is this thing called pain? *J. Clin. Invest.* 120 3742–3744. 10.1172/JCI45178 21041955 PMC2965006

[B55] Yoshimura M. Furue H. (2006). Mechanisms for the anti-nociceptive actions of the descending noradrenergic and serotonergic systems in the spinal cord. *J. Pharmacol. Sci.* 101 107–117. 10.1254/jphs.CRJ06008X 16766858

[B56] Zhang H. Zhao Y. Wang Z. (2015). Chronic corticosterone exposure reduces hippocampal astrocyte structural plasticity and induces hippocampal atrophy in mice. *Neurosci. Lett.* 592 76–81. 10.1016/j.neulet.2015.03.006 25748318

[B57] Zhang Z. J. Cao D. L. Zhang X. Ji R. R. Gao Y. J. (2013). Chemokine contribution to neuropathic pain: respective induction of CXCL1 and CXCR2 in spinal cord astrocytes and neurons. *Pain* 154 2185–2197. 10.1016/j.pain.2013.07.002 23831863 PMC3899784

