# Supplementary material for: Astrocyte Activation in Locus Coeruleus Is Involved in Neuropathic Pain Exacerbation Mediated by Maternal Separation and Social Isolation Stress
Source: Front Pharmacol. 2017 Jun 28;8:401. doi: 10.3389/fphar.2017.00401 (PMC5487383; doi:10.3389/fphar.2017.00401)

***Frontiers in Pharmacology***

***Supplementary information***

**Astrocyte Activation in Locus Coeruleus is involved in Neuropathic Pain exacerbation mediated by Maternal Separation and Social Isolation Stress**

**◯Kazuo Nakamoto^1^, Fuka Aizawa^1^, Megumi Kinoshita^1^, Yutaka Koyama^2^, Shogo Tokuyama^1^***

**^1^Department of Clinical Pharmacy, School of Pharmaceutical Sciences, Kobe Gakuin University, 1-1-3 Minatojima, Chuo-ku, Kobe 650-8586, Japan, ^2^Laboratory of Pharmacology, Faculty of Pharmacy, Osaka Ohtani University, 3-11-1 Nishikiori-Kita, Tondabayashi, Osaka 584-8540, Japan**

*** Correspondence:**

**Department of Clinical Pharmacy, School of Pharmaceutical Sciences, Kobe Gakuin University, 1-1-3 Minatojima, Chuo-ku, Kobe 650-8586, Japan, Tel: +81-78-974-1551, Fax: +81-78-974-4780, E-mail: stoku@pharm.kobegakuin.ac.jp (S. Tokuyama)**

***Methods***

**Western blot analysis**

Western blotting was performed as previously described, with some modifications (Nishinaka et al., 2015). Each brain region was identified according to the atlas of Franklin and Paxinos (Franklin and Paxinos, 2008). Each tissue sample was homogenized in homogenization buffer including the several protease inhibitors and phosphatase inhibitors. Protein samples (10 μg/lane) were separated using sodium dodecyl sulfate-polyacrylamide gel (7.5%) electrophoresis and transferred to nitrocellulose membranes (Bio-Rad Laboratories, Inc. Hercules, CA, USA). Membranes were incubated overnight at 4 °C with polyclonal anti-GFAP (1:1000) (Chemicon, CA, U.S.A.) or anti-glyceraldehyde-3-phosphate dehydrogenase (GAPDH) (1:20,000) (Chemicon International, Temecula, CA, USA) primary antibodies. The membranes were incubated with secondary antibodies, horseradish peroxidase-conjugated anti-mouse IgG (1:1,000) (Milipore, Billerica, MA, U.S.A.) for GFAP and anti-mouse IgG (1:10,000) (KPL, Guildford, UK) for GAPDH, for 1 h at room temperature. Immunoreactive bands were detected using the enhanced chemiluminescence Western blotting analysis system (GE Healthcare, Buckinghamshire, UK) and visualized using a light-capture system (AE-6981) (ATTO Corp., Tokyo, Japan).

**Results**

**GFAP protein expression did not change in MSSI stress treated male mice after PSL**

GFAP protein expression did not change in the medulla oblongata, hippocampus, midbrain, prefrontal cortex and hypothalamus of MSSI stress treated male mice after PSL (Supple Fig. 1).

**GFAP protein expression did not change in MSSI stress treated female mice after PSL**

GFAP protein expression showed tendency of increase in the medulla oblongata of MSSI stress treated female mice after PSL. However, GFAP protein expression did not change in other brain area such as the hippocampus, midbrain, prefrontal cortex and hypothalamus of MSSI stress treated female mice after PSL (Supple Fig. 2).

**Figure legend**

**Supplementary Figure 1**

**GFAP protein expression did not change in MSSI stress treated male mice after PSL**

(A) Medulla oblongata: n=6. (B) Hippocampus: n=6. (C) Midbrain: n=6 (D) Prefrontal cortex: n=6 (E) Hypothalamus: n=6. Panel represents a representative western immunoblot of GFAP (50 kDa) and GAPDH (37 kDa). The results are expressed as percentage of Control Sham. Date are shown as the mean ± S.E.M.

**Supplementary Figure 2**

**GFAP protein expression did not change in MSSI stress treated female mice after PSL**

(A) Medulla oblongata: n=9. (B) Hippocampus: n=6. (C) Midbrain: n=6 (D) Prefrontal cortex: n=6 (E) Hypothalamus: n=6. Panel represents a representative western immunoblot of GFAP (50 kDa) and GAPDH (37 kDa). The results are expressed as percentage of Control Sham. Date are shown as the mean ± S.E.M.

**References**

Franklin KBJ, Paxinos G (2008). The MOUSE BRAIN IN STEREOTAXIC COORDINATES COMPACT THIRD EDITION ACADEMIC PRESS: 1-242.

Nishinaka, T., Kinoshita, M., Nakamoto, K., Tokuyama, S., (2015b). Sex differences in depression-like behavior after nerve injury are associated with differential changes in brain-derived neurotrophic factor levels in mice subjected to early life stress. *Neurosci. Lett.* 592, 32–36. doi: 10.1016/j.neulet.2015.02.053.

Supplementary Figure 1


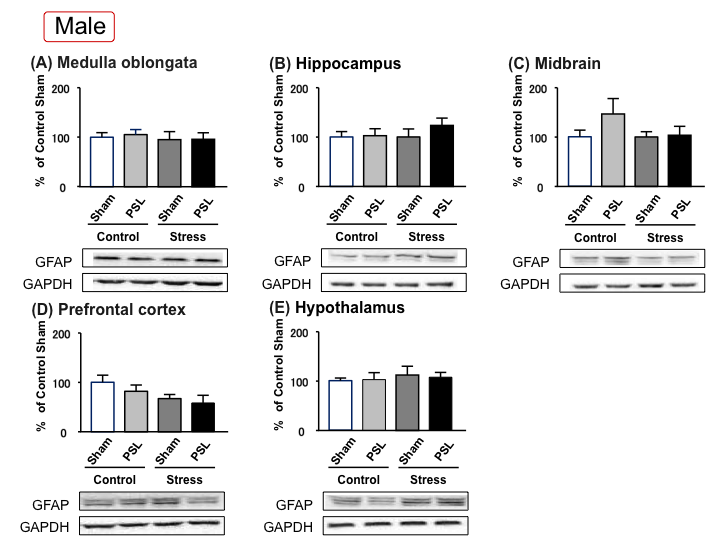


Supplementary Figure 2


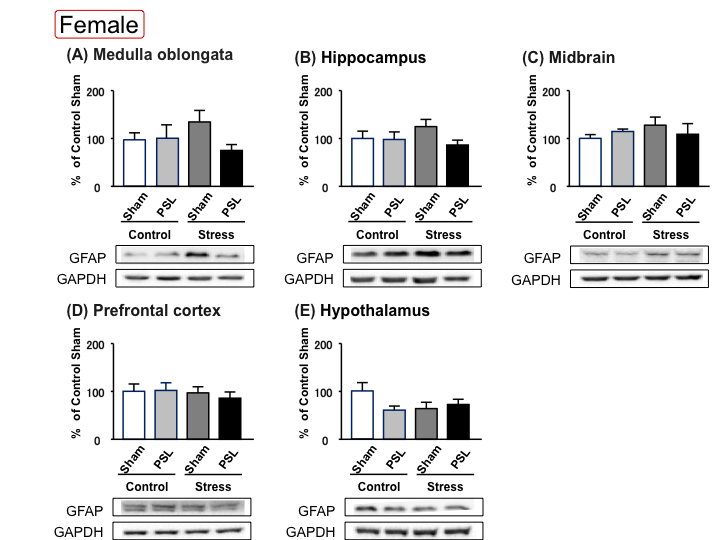

Supplement: Supplementary file 1 [file Data_Sheet_1.DOCX]
